# Metagenomics of a Photo-Fermentative Bacterial Solution and Its Effect on the Growth And Yield of Mini Green Cos Lettuce

**DOI:** 10.21315/tlsr2026.37.1.5

**Published:** 2026-03-31

**Authors:** Nanthavut Niyomvong, Duanpen Wongsorn, Nittaya Pitiwittayakul

**Affiliations:** 1 Science Center, Faculty of Science and Technology, Nakhon Sawan Rajabhat University, Nakhon Sawan 60000, Thailand; 2 Department of Biology and Biotechnology, Faculty of Science and Technology, Nakhon Sawan Rajabhat University, Nakhon Sawan 60000, Thailand; 3 Department of Plant Science, Faculty of Agricultural Innovation and Technology, Rajamangala University of Technology Isan, Nakhon Ratchasima Campus, Nakhon Ratchasima 30000, Thailand

**Keywords:** Biofertiliser, Metagenomics, Photosynthetic Bacteria, Plant Growth Promotion, *Rhodopseudomonas*

## Abstract

Photosynthetic bacteria (PSB) are widely utilised in agriculture to enhance plant growth and crop quality by improving nutrient uptake and phytohormone production. This study aimed to analyse the metagenomic composition of a photo-fermentative bacterial solution derived from fermentation and assess its effects on the growth and yield of Mini Green Cos lettuce. Metagenomic analysis revealed that *Bacteroidota* (38%) was the most abundant phylum, followed by *Proteobacteria* (23%), *Thermotogota* (17%) and *Firmicutes* (15%). Within *Proteobacteria*, *Alphaproteobacteria* was dominant followed by *Gammaproteobacteria*. At the genus level, *Petrimonas* (22%), uncultured clones belonging to family *Petrotogaceae* (17%), *Rhodopseudomonas* (11%), *Rubrivivax* (6%), and an unidentified genus from *Lentimicrobiaceae* (4%) were the most prevalent. These findings highlight the microbial diversity of PSB solution, suggesting its potential role in plant growth promotion. A plant growth experiment was conducted using a Completely Randomised Design (CRD) with four treatments: control (T1), chemical fertiliser (T2), undiluted PSB solution (T3) and PSB solution diluted at a 1:1 ratio (T4), with 10 replicates per treatment. Among all treatments, lettuce irrigated with undiluted PSB solution (T3) exhibited the highest growth rate, yield and total chlorophyll content. However, its performance was not significantly different from that of the chemical fertiliser treatment (T2). These results suggest that PSB can effectively promote plant growth and yield, yielding results comparable to chemical fertilisers. Therefore, photo-fermentative bacterial solutions offer a sustainable and eco-friendly alternative to chemical fertilisers, supporting environmentally conscious agricultural practices.

**HIGHLIGHTS**

- Taxonomic analysis revealed a diverse bacterial community in the fermented photosynthetic bacterial (PSB) solution, dominated by *Bacteroidota*, followed by *Proteobacteria*, *Thermotogota* and *Firmicutes*.
- Metagenomic analysis revealed a diverse microbiome in the fermented PSB solution, dominated by *Rhodopseudomonas* and enriched with obligate anaerobes such as *Petrimonas*, *Petrotogaceae* AUTHM297, *Wolinella succinogenes* and *Clostridium tertium*.
- The PSB solution effectively enhanced the growth and yield of mini green cos lettuce, with plant weights comparable to those obtained using chemical fertilisers.

## INTRODUCTION

Agriculture accounts for approximately one-third of the world’s gross domestic product (GDP). However, with the global population projected to reach 9.5 billion by 2050, the food demand is expected to increase by approximately 60% (Kumar *et al*. 2022; Ammar *et al*. 2023). To meet this demand, chemical fertilisers and pesticides are widely used to increase crop productivity. Nonetheless, the prolonged use of chemical fertilisers contributes to the reduction in soil organic matter and the deterioration of agricultural soil quality. Overuse of chemical fertilisers results in hardened soil, decreased fertility and pollution of air, water and soil, as well as a reduction in vital soil and mineral nutrients, all of which pose environmental risks (Pahalvi *et al*. 2021). Furthermore, the chemical fertiliser required for a 321 million-ton crop yield by 2020 was 28.8 million tons. However, only 21.6 million tons were available, resulting in a shortage of 7.2 million tons, which significantly increased costs for smallholder farmers (Mehata *et al*. 2023). As a sustainable alternative, biofertilisers offer an eco-friendly solution to reducing reliance on chemical fertiliser (Chakraborty & Akhtar 2021). Biofertilisers are defined as substances that contain living microorganisms, which colonise the rhizosphere or plant tissues and promote growth by increasing the supply or availability of primary nutrients to the target crops when applied to soils, seeds, or plant surfaces (Santhosh Kumar *et al*. 2018. Among these, phototrophic microorganisms known as purple non-sulfur bacteria are gaining significance in plant production due to their ability to synthesise and accumulate valuable chemicals that support plant growth. Notable characteristics of purple non-sulfur bacteria include the accumulation of polyphosphate, the synthesis of pigments and vitamins, and the production of chemicals that promote plant growth (Sakarika *et al*. 2020). Therefore, the objectives of this study were to prepare PSB solution and investigate its bacterial community through metagenomic analysis. Moreover, the effect of PSB solution on the growth and yield of mini–Green Cos lettuce was characterised.

## MATERIALS AND METHODS

### Preparation of PSB Solution

**FIGURE 1:**
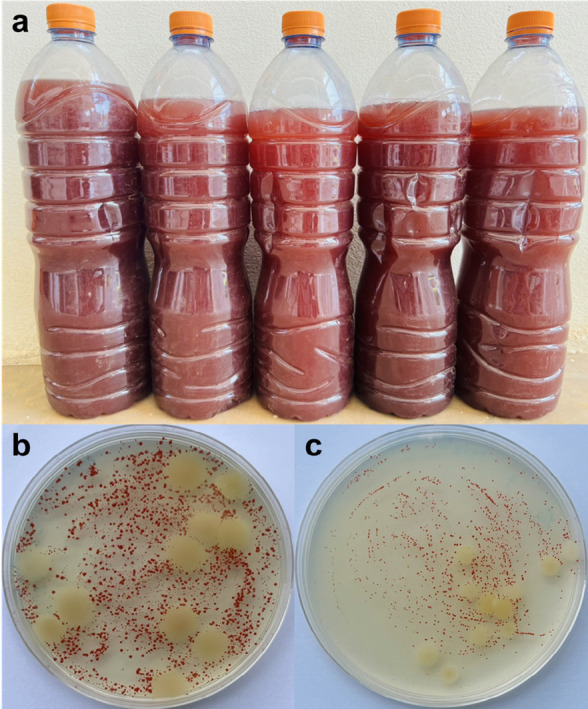
(a) The appearance of the PSB solution after 14 days of natural fermentation, culturable bacterial colonies in PSB solutions on (b) PCA medium, and on (c) RCVBN medium.

The PSB solution was prepared following a modified method adapted from Synrem *et al*. (2018). Briefly, 1.4 L of chlorine-free natural water (groundwater), 50 mL of PSB solution (inoculum from natural fermentation) and 30 mL of a mixture consisting of beaten chicken eggs (2 eggs), monosodium glutamate (15 g) and fish sauce (15 mL) were combined in 1.5 L water bottles. The bottles were tightly sealed with caps, mixed and stored in a well-lit area away from sunlight for two weeks until the pale-yellow liquid turned red (Fig. 1a). The bottles were shaken once a week. Many bottles of PSB solutions were prepared to ensure a sufficient quantity for use in the plant growth test. After 7 days–14 days of cultivation, all bottles of PSB solutions were pooled together in a large tank, from which a sample was taken for metagenomic extraction and applied to lettuce cultivation.

### Analysis of Chemical Properties and Bacterial Counts in the PSB Solution

Available nitrogen of PSB solution was analysed using the Kjeldahl method (Sáez-Plaza *et al*. 2013). Total phosphorus and potassium were determined through wet digestion. Phosphorus in the extract was detected using phosphomolybdic acid and measured using a spectrophotometer at 660 nm (Malá & Lagová 2014). Total potassium was detected using an atomic absorption spectrophotometer (David 1960). Table 1 shows the chemical properties of the PSB solution used in the experiment.

In this study, the optical density (OD) of the PSB solutions in all sampling bottles and in the pooled large tank was approximately 4 at 600 nm. The bacterial concentration in the PSB solutions was approximately 10^7^–10^8^ colony-forming units (CFU), as determined by total plate count analysis using Plate Count Agar (PCA) (Fig. 1b) and RCVBN media (Fig. 1c) for PSB cultivation (Zhao *et al*. 2025a). According to Lo *et al*. (2020), the OD600 and CFUs values were positively correlated during the fermentation. The most dominant culturable bacteria were purple non-sulfur bacteria, which formed red colonies on both agar media, consistent with the results from metagenomic analysis.

### Bacterial Community Analysis

#### Metagenomic extraction

Metagenomic DNA was extracted from the bacterial community in PSB solution using the QIAamp PowerFecal Pro DNA kit (Qiagen, Hilden, Germany) according to the manufacturer’s protocols.

#### 16S rRNA library sequencing

The prokaryotic 16S rRNA gene at the V3V4 region was performed using the Qiagen QIAseq 16S/ITS Region panel (Qiagen, Hilden, Germany). The targeted PCR cycling conditions were as follows: an initial denaturation at 95°C for 2 min, followed by 12 cycles at 95°C for 30 sec, 50°C for 30 sec and 72°C for 2 min, with a final extension at 72°C for 7 min. The 16S rRNA amplicons were purified using QIAseq magnetic beads and subsequently labelled with different sequencing adaptors using QIAseq 16S/ITS Region Panel Sample Index PCR Reaction (Qiagen, Hilden, Germany). The index PCR reaction cycling conditions were as follows: initial denaturation at 95°C for 2 min, followed by 19 cycles of denaturation at 95°C for 30 sec, 60°C for 30 sec and 72°C for 2 min, with a final extension at 72°C for 7 min. The DNA libraries with different indexes (approximately 630 bp) were purified using QIAseq magnetic beads (Qiagen, Hilden, Germany). The quality and quantity of DNA libraries were evaluated using DeNovix QFX Fluorometer and QIAxcel Advanced (Qiagen, Hilden, Germany), respectively. Paired-end sequencing, 2 × 300, was performed using Illumina Miseq platform following the manufacturer’s protocols (Illumina, San Diego, CA, USA).

#### Bioinformatics analysis and taxonomic analysis of amplicon 16S-rRNA data

The raw sequences were categorised into groups based on their 5′ barcode sequences and processed using DADA2 v1.16.0 pipeline (https://benjjneb.github.io/dada2/). The DADA2 pipeline characterises microbial diversity and community structures by identifying unique amplicon sequence variants (ASVs). Microbial taxa were classified using the Silva version 138 as a reference database. The alpha diversity calculations were performed using Phyloseq (v1.38.0). The metagenome sequence data have been submitted to the NCBI SRA under BioProject number PRJNA1244289.

#### Prediction of functional gene content

Each BIOM-format table was used as input for PICRUSt2 (Douglas *et al*. 2020) and the Kyoto Encyclopedia of Genes and Genomes (KEGG) database (Kanehisa & Goto 2000) was used to predict the functional gene content. PICRUSt2 was run with the default cutoff nearest sequenced taxon index (NSTI) of 2.0 and functions from the resulting prediction table were transformed to relative abundances.

#### Evaluating the growth and determination of agronomic properties of lettuce

Mini green cos lettuce seeds were sown in a planting tray filled with non-sterile peat moss. The lettuce seedlings were transferred to individual pots after 3 weeks of planting. The experiment included four treatment groups: T1: Negative control (water), T2: Chemical fertiliser, T3: undiluted PSB solution and T4: PSB solution diluted with water at a 1:1 ratio. Each treatment was performed in 10 replicates. The treatments for the lettuce were applied as follows: T1: watered with 200 mL per pot daily, T2: watered with 200 mL daily and fertilised once a week with a 46–0–0 formula dissolved in water at a 1:100 ratio, T3: watered daily with 200 mL of undiluted PSB solution, and T4: watered daily with 200 mL PSB solution diluted at a 1:1 ratio with water. Plant height and number of leaves were recorded every week until harvest. After 56 days of cultivation, the plants were carefully uprooted, with excess soil removed by gentle shaking, followed by rinsing with distilled water. Surface moisture was removed using absorbent paper. The agronomic properties of the lettuce were measured, including leaf area, fresh weight and dry weight of both aboveground and underground plant parts.

#### Chlorophyll content of lettuce leaves

The chlorophyll content in mini green cos lettuce leaves was determined using the method described by Vafadar *et al*. (2014). Leaf samples from four different treatments, with three replicates per treatment, were collected and processed. Approximately 100 mg of leaf tissue, excluding major veins and leaf edges, was finely ground using a mortar and pestle. The ground tissue was extracted with 20 mL of acetone and filtered through cheesecloth to remove debris. The final extract volume was adjusted to 30 mL with acetone. The chlorophyll extract was stored in a light-protected glass container wrapped in aluminum foil to prevent photodegradation.

The absorbance of the acetone-extracted chlorophyll solution was measured at 645 nm and 663 nm using a spectrophotometer. The chlorophyll a and b concentrations were calculated using the following equations:


$$\begin{array}{l} \text{Chlorophyll a }(\text{mg per }100\;\text{g})=[12.7\;(\text{OD}663)-2.69\;(\text{OD}645)]\times \text{v}/(1,000\times \text{w})\\ \text{Chlorophyll b }(\text{mg per }100\;\text{g})=[22.9\;(\text{OD}645)-4.68\;(\text{OD}663)]\times \text{v}/(1,000\times \text{w}) \end{array}$$

where v is the final volume of the extract (mL), w is the weight of the leaf sample (g) and OD represents the absorbance at the specified wavelengths.

### Statistical Analysis

Statistical analysis was performed using the Statistical Analysis System (SAS) software (version 9, SAS Institute Inc). Mean differences among treatments were compared using analysis of variance (ANOVA) followed by Duncan’s Multiple Range Test (DMRT) at a 95% confidence level.

## RESULTS

### Metagenomic Analysis of PSB Solution

**FIGURE 2:**
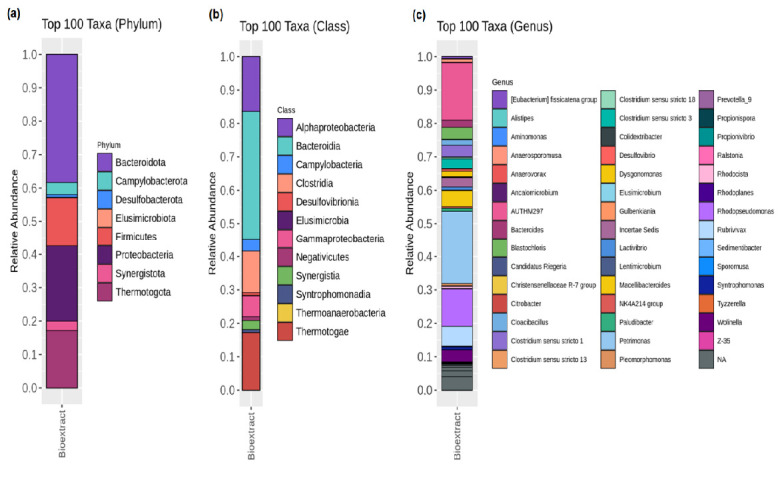
The relative abundances of microbes annotated for the 16S rRNA amplicon datasets at the (a) phylum, (b) class and (c) genus levels of PSB solution.

**FIGURE 3:**
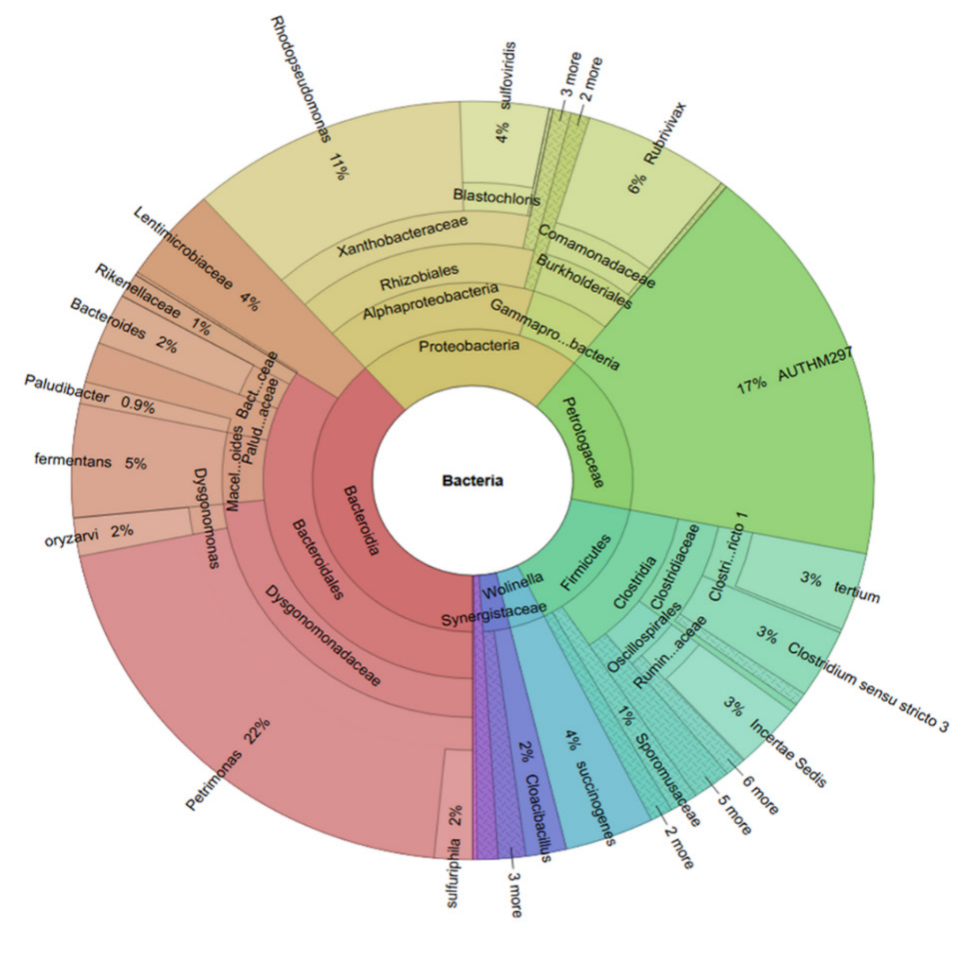
Krona chart showing the distribution of the most abundant bacterial family *Dysgonomonadaceae, Petrotogaceae, Xanthobacteraceae, Comamonadaceae* and *Lentimicrobiaceae* and in the genus *Petrimonas*, AUTHM297, *Rhodopseudomonas* and *Rubrivivax* in the PSB solution. The Krona chart was created using Krona open-source software (Ondov *et al*. 2021).

The PSB solution was cultured for 7 days–14 days, resulting in a deep red suspension (Fig. 1) that was used for plant treatment. To gain scientific insights into the bacterial community, the PSB solution was analysed using Illumina-Miseq high-throughput sequencing targeting the V3–V4 region of the 16S rRNA gene. The alpha diversity indices, including observed species, Chao1, Shannon and Simpson, which reflect species richness and evenness, are presented in Table 2. A total of 125 taxa were identified at the phylum level. The taxonomy classification results revealed the presence of various bacterial phyla. At the phylum level, *Bacteroidota, Thermotogota, Proteobacteria, Campylobacterota, Firmicutes, Synergistota, Desulfobacterota* and *Elusimicrobiota* were detected (Fig. 2). Among these, *Bacteroidota* (38%) was the most abundant in the PSB solution, followed by *Proteobacteria* (23%), *Thermotogota* (17%) and *Firmicutes* (15%) (Fig. 2a). At the class level, *Alphaproteobacteria* and *Gammaproteobacter* accounted for approximately 16% and the remaining proportion, respectively (Fig. 2b). At the genus level (Fig. 2c), the five most dominant genera in the PSB solution were *Petrimonas* (22%), uncultured clones, AUTHM297 (belonging to family *Petrotogaceae*, phylum *Thermotogae* (17%), *Rhodopseudomonas* (11%), *Rubrivivax* (6%) and an unidentified genus of family *Lentimicrobiaceae* (4%) (Fig. 3).

The KEGG Orthology (KO) database was utilised to infer the functional profiles of genes associated with plant growth-promoting traits (Table 3). The *trp* operon, which is associated with auxin synthesis, was identified. Genes associated with siderophore and aminolevulinic acid production were also detected. Several *nif* genes involved in nitrogen fixation—including *nifA, nifB, nifD, nifE, nifF, nifH, nifJ, nifK, nifN, nifQ, nifT*, *nifU, nifV, nifW, nifX* and *nifZ*—were also identified. Additionally, genes involved in phosphate solubilisation within phosphorus metabolism such as *pstA, pstB, pstC* and *pstS*, along with components of the PHO regulon, were observed. Several of the most abundant bacterial genera identified in this study have been previously reported to exhibit plant growth-promoting functions, as detailed in Table 4.

### Growth of Mini Green Cos Lettuce

The growth performance of mini green cos lettuce was evaluated across four treatments: control group (T1), treatments with chemical fertiliser (T2), undiluted PSB solution (T3) and PSB solution diluted at a 1:1 with sterile distilled water (T4). Growth assessments were conducted by measuring plant height and counting the number of leaves over a period of five weeks.

**FIGURE 4:**
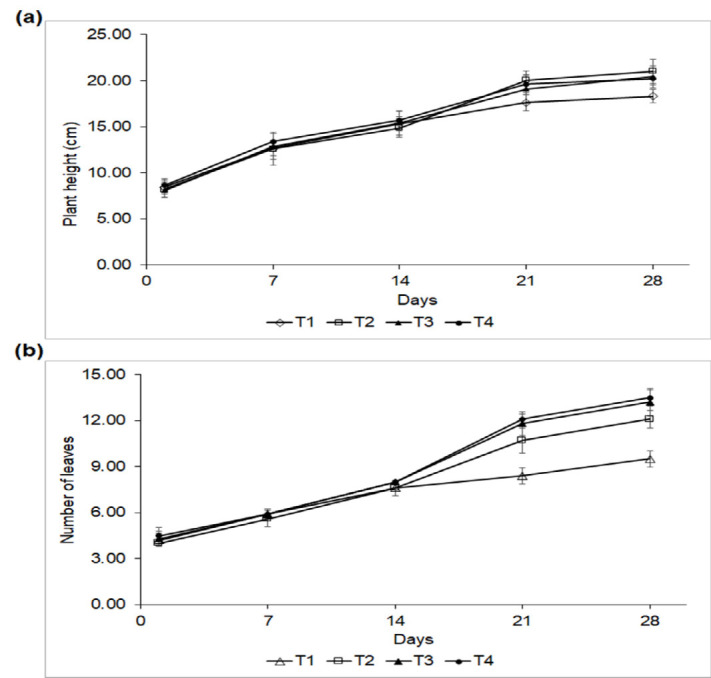
(a) Plant height and (b) number of leaves of lettuce under different treatments. T1, control; T2, chemical fertiliser; T3, undiluted PSB solution; and T4, PSB solution diluted with distilled water at a 1:1 ratio.

During the first week, plant height and leaf number were similar across all treatments. However, by weeks 4 and 5, T2, T3 and T4 showed comparable plant heights which were significantly different from those of the control group (*P* < 0.0001) (Fig. 4a). At week 5, the plant heights for T2, T3, T4 and T1 were 21.01 cm, 20.40 cm, 20.22 cm and 18.30 cm, respectively. In terms of leaf count, by weeks 4 and 5, T3 and T4 had the highest number of leaves, which were significantly different from the other treatments (*P* < 0.0001), followed by T2 and T1, respectively (Fig. 4b).

### Yield of Mini Green Cos Lettuce

**FIGURE 5:**
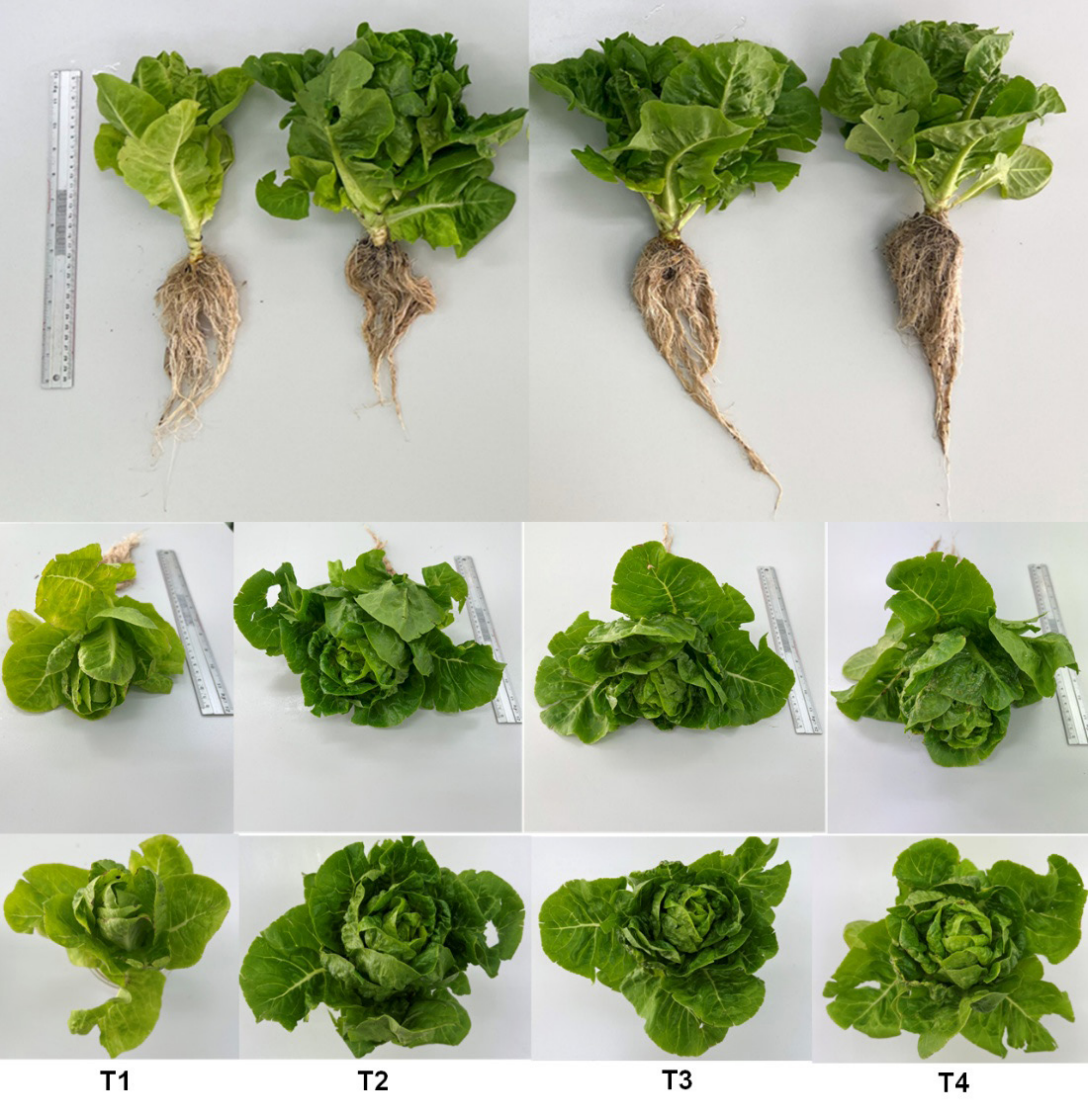
Growth of mini green cos lettuce cultured for 56 days under different treatments. T1 = control; T2 = chemical fertiliser; T3 = undiluted PSB solution and T4 = PSB solution diluted with distilled water at a 1:1 ratio.

The effects of PSB on the growth and yield of mini green cos lettuce are shown in Table 5 and Fig. 5. After 56 days of cultivation, T3 exhibited the highest fresh weight at 253.91 g, which was not significantly different from T2 at 244.78 g. T4 yielded a fresh weight of 179.93 g, which was significantly lower than both T2 and T3. Dry weight results exhibited a similar pattern to that of fresh weight across all treatments. The dry weight of T2 and T3 were 7.55 g and 7.54 g, respectively, with no significant difference between them. These values were significantly higher than those of T4 and T1. In contrast, T4 yielded the highest root fresh weight (43.70 g), significantly surpassing all other treatments. Similarly, T4 had the highest root dry weight at 5.13 g, which was significantly higher than those of the T2, T3 and T1. Leaf area measurements, taken from the fourth leaf from the base across all treatments, showed that the T4 exhibited the largest leaf area (614.07 cm^2^). However, it was not statistically different from other treatments.

### Chlorophyll Content

The chlorophyll content of lettuce leaves was determined on the harvest day (56 days after cultivation). Chlorophyll a content was highest in T3 (195.06 mg/100 g), showing no significant difference from T2 but significantly higher than both T1 and T4 (Table 6). Similarly, T3 also showed the highest chlorophyll b content (164.08 mg/100 g), though not significantly different from T4. For total chlorophyll, T3 significantly outperformed all other treatments, while T2 and T4 did not differ significantly from each other.

## DISCUSSION

The terms biofertiliser and bioinoculant have emerged from the significant advancements in research on plant-microorganism interactions. A biofertiliser is typically described as a substance containing living microorganisms that, when applied to seeds, plant surfaces or soil, colonise the plant’s rhizosphere or interior, enhancing growth by improving the supply or availability of essential nutrients to the host plant (Basu *et al*. 2021). Among biofertilisers, PSB have gained attention as sustainable alternatives to chemical fertilisers. This study utilised natural fermentation process involving locally available ingredients (eggs, fish sauce, monosodium glutamate and natural chlorine-free water) to cultivate beneficial PSB. Microbial community analysis showed that the PSB solution primarily consisted of the dominant purple non-sulfur bacteria, *Rhodopseudomonas* sp., followed by *Rubrivivax* sp. and *Blastochloris sulfoviridis*. These PSB have been reported to promote plant growth through several mechanisms, such as phytohormone production, nutrient solubilisation, nitrogen fixation, siderophore production and stress tolerance enhancement through the production of volatile organic compounds that inhibit fungal pathogens (Sakpirom *et al*. 2017; Zhang *et al*. 2019; Maeda 2022; Xuan *et al*. 2024; Khuong *et al*. 2020; Zhao *et al*. 2025b; Lee *et al*. 2021). Surachat *et al*. (2022) reported genes associated with plant growth promotion in *Rhodopseudomonas* strains, including *nif* operon associated with nitrogen fixation, *pst* operon for phosphate solubilisation within phosphorus metabolism subsystem. These findings are consistent with the results of this study. According to the KEGG database categories, functional analysis of the PSB solution predicted gene functions associated with plant growth-promoting traits, including IAA production, phosphate solubilisation, siderophore (Pakarian & Pawelek 2016) and aminolevulinic production (Jiang *et al*. 2022). Moreover, the metabolic components of the PSB solution have been reported through LC-MS analysis, revealing a variety of amino acids and vitamins, and growth-promoting hormones, including 5-aminosalicyclic acid, indole-3-acetic acid and 5-aminolevulinic acid (Du *et al*. 2023). Due to the natural fermentation process used to obtain the PSB solution, other non-PSB microorganisms were also present in addition to PSB in this study. The dominant obligate anaerobic bacteria identified included *Petrimonas*, *Petrotogaceae* AUTHM297, *Wolinella succinogenes*, *Clostridium sensu stricto 1 tertium*, *Ruminococcaceae Incertae Sedis* and *Macellibacteroides fermentans*. These bacteria can produce short-chain fatty acids, such as acetic, propionic and butyric acids, through glucose fermentation or protein hydrolysis and amino acid metabolism (Jabari *et al*. 2012; Jung *et al*. 2023). This process contributes to the production of short-chain fatty acids, which can enhance PSB growth by serving as nutrients or promoting plant growth through the synthesis of growth-enhancing compounds such as *n*-caproate (Liu *et al*. 2020; Kantha *et al*. 2012; Smith *et al*. 2023).

In this study, the analysis revealed that treatment T3 (PSB solution) showed no statistically significant difference in plant height or shoot fresh weight compared with T2 (chemical fertiliser). Moreover, some parameters such as leaf numbers and total chlorophyll content, were significantly higher than in T2. The results of this study are consistent with previous findings on the effect of PSB on the physiological parameters of leafy vegetables (Wong *et al*. 2014; Hsu *et al*. 2021). Furthermore, the findings of this study align with those of Synrem *et al*. (2018), who investigated the effects of using a combination of PSB and biochar. Their study demonstrated that applying PSB and biochar significantly improved lettuce growth parameters, including fresh and dry biomass, root weight, leaf area and chlorophyll content, compared to control treatments. The most effective treatment combined 40 mL of PSB with 10 L of water and 5 t/ha of biochar, resulting in the highest growth and yield metrics. Similarly, Jamir *et al*. (2020), demonstrated that the application of PSB and biochar at different concentrations increased fresh and dry biomass, root dry weight, leaf area, and chlorophyll content of broccoli compared to the control. Additionally, *Rhodopseudomonas palustris* PS3, a PSB strain, has been reported to promote the growth of non-heading Chinese cabbage (*Brassica rapa* var. *chinensis*) by enhancing nitrate uptake and stimulating the accumulation of endogenous auxin in young expanding leaves. This process increases the proliferation of leaf cells during leaf development (Hsu *et al*. 2021). Yen *et al*. (2022) also concluded that the foliar application of PSB, *Rhodopseudomonas palustris*, to rice significantly increased tiller number, leaf chlorophyll content and lodging resistance, including root length, root dry weight, productive tillers per plant, average grains per plant, grain yield and harvest index.

Lipopolysaccharide (LPS), a component of Gram-negative bacterial cell wall, has been identified as a key factor in promoting plant growth. Notably, Hayashi *et al*. (2022) demonstrated that foliar application of *R. sphaeroides* LPS at concentrations ranging from 10 pg/mL to 100 pg/mL significantly enhanced growth in *Brassica rapa* var. *perviridis* (komatsuna). According to Iwai *et al*. (2023), LPS functions as an active component of purple non sulfur bacteria in plants, as supported by gene expression data from rice (*Oryza sativa*) seedling. Despite their nonpathogenic nature, PSB stimulate plant defense responses at the gene expression level by upregulating genes involved in the jasmonic acid (JA) signaling pathway and those associated with biotic and abiotic stress tolerances.

Based on the findings of this study, the PSB solution produced through natural fermentation effectively promotes the growth and yield of mini green cos lettuce. Notably, plant weight did not differ significantly between PSB-treated and chemical fertiliser-treated groups. It can be concluded that PSB solution has potential as a biofertiliser to reduce the use of chemical fertilisers.

## CONCLUSION

Metagenomic analysis revealed a diverse microbial composition within the naturally fermented PSB solution. The dominant purple non-sulfur bacteria identified were *Rhodopseudomonas* sp. Additionally, several non-PSB, obligate anaerobic bacteria were prevalent, including *Petrimonas*, *Petrotogaceae* AUTHM297, *Wolinella succinogenes* and *Clostridium sensu stricto 1 tertium*. Application of undiluted PSB enhanced the growth, yield and chlorophyll a content of mini green cos lettuce, performing comparably to chemical fertilisers. These findings suggest that photo-fermentative bacterial solutions offer a sustainable, eco-friendly alternative to chemical fertilisers, supporting environmentally friendly agricultural practices.

## Figures and Tables

**TABLE 1:** Chemical properties of PSB solutions.

Sample	Chemical properties				

	pH	EC (dS/m)	Total N (%)	Total P (mg/L)	Total K (mg/L)
PSB solution	6.195 ± 0.007	3.780 ± 0.042	0.060 ± 0.000	62.890 ± 1.103	116.865 ± 2.623

**TABLE 2:** Diversity indices of the microbial communities obtained by the 16S rRNA amplicon analysis of PSB solution.

Sample	Chao1	Standard error of Chao1	Shannon	Simpson
PSB solution	126.00	1.58	2.95	0.90

**TABLE 3:** Predictive functional gene profiling of microbial communities associated with plant growth promotion.

Plant-growth promotion traits	KEGG orthology	Genes	Function	Taxon function abundance
IAA production	K01695	*trpA*	tryptophan synthase alpha chain	58980.93
	K01696	*trpB*	tryptophan synthase beta chain	59069.53
	K01609	*trpC*	indole-3-glycerol phosphate synthase	54273.10
	K00766	*trpD*	anthranilate phosphoribosyltransferase	38239.43
	K01657	*trpE*	anthranilate synthase component I	47833.43
	K01817	*trpF*	phosphoribosylanthranilate isomerase	59579.05
	K01658	*trpG*	anthranilate synthase component II	36911.10
	K03720	*trpR*	TrpR family transcriptional regulator, *trp* operon repressor	4.50
	K01867	*trpS*	tryptophanyl-tRNA synthetase	66447.72

Nitrogen fixation	K02584	*nifA*	Nif-specific regulatory protein	18580.50
	K02585	*nifB*	nitrogen fixation protein NifB	18361.50
	K02586	*nifD*	nitrogenase molybdenum-iron protein alpha chain	24156.50
	K02587	*nifE*	nitrogenase molybdenum-cofactor synthesis protein NifE	14252.00
	K03839	*nifF*	flavodoxin I	41395.00
	K02588	*nifH*	nitrogenase iron protein NifH	41461.60
	K03737	*nifJ*	pyruvate-ferredoxin/flavodoxin oxidoreductase	55830.88
	K02591	*nifK*	nitrogenase molybdenum-iron protein beta chain	28371.50
	K02592	*nifN*	nitrogenase molybdenum-iron protein NifN	13847.50
	K15790	*nifQ*	nitrogen fixation protein NifQ	12671.50
	K02593	*nifT*	nitrogen fixation protein NifT	12671.50
	K04488	*nifU*	nitrogen fixation protein NifU and related proteins	46688.22

	K02594	*nifV*	homocitrate synthase NifV	18308.00
	K02595	*nifW*	nitrogenase-stabilising/protective protein	12671.50
	K02596	*nifX*	nitrogen fixation protein NifX	12671.50
	K02597	*nifZ*	nitrogen fixation protein NifZ	23115.50

Aminolevulinic acid	K09698	*gltX*	nondiscriminating glutamyl-tRNA synthetase	17148.50
	K02492	*hemA*	glutamyl-tRNA reductase	17373.39
	K01845	*hemL*	glutamate-1-semialdehyde 2,1-aminomutase	27737.39

Siderophore	K00216	*entA*	2,3-dihydro-2,3-dihydroxybenzoate dehydrogenase	10.50
	K01252	*entB*	bifunctional isochorismate lyase / aryl carrier protein	10.50
	K02361	*entC*	isochorismate synthase	20814.00
	K02364	*entF*	enterobactin synthetase component F	10.50
	K08225	*entS*	MFS transporter, ENTS family, enterobactin (siderophore) exporter	4.50

Phosphate solubilisation and transport gene	K01077	*phoA, phoB*	alkaline phosphatase	80119.00
	K01113	*phoD*	alkaline phosphatase D	5796.00
	K09474	*phoN*	acid phosphatase class A	9.00
	K07637	*phoQ*	sensor histidine kinase PhoQ	10.50
	K07636	*phoR*	phosphate regulon sensor histidine kinase PhoR	80421.89
	K02039	*phoU*	phosphate transport system protein	67933.72
	K02038	*pstA*	phosphate transport system permease protein	59075.32
	K02036	*pstB*	phosphate transport system ATP-binding protein	59075.32
	K02037	*pstC*	phosphate transport system permease protein	56656.82
	K02040	*pstS*	phosphate transport system substrate-binding protein	64452.32
	K00117	*gcd*	quinoprotein glucose dehydrogenase	11361.50
	K03788	*aphA*	acid phosphatase class B	4.50
	K09612	*iap*	alkaline phosphatase isozyme conversion protein	4.50
	K06019	*ppaX*	pyrophosphatase PpaX	10089.00
	K01595	*ppc*	phosphoenolpyruvate carboxylase	20546.83
	K00937	*ppk*	polyphosphate kinase	39945.43

**TABLE 4:** Major bacterial communities that play an essential role in plant growth promotion in PSB solutions.

Phylum	Family	Bacteria (Genus)	Function (Characteristics)	References
*Bacteroidota*	*Dysgonomonadaceae*	*Petrimonas*	Bacteroidetes is a diverse group of species capable of fermenting sugars, hydrolysing proteins and polysaccharides, and producing acids such as butyric, propionic, succinic and acetic acids. They produce CO(2), hydrogen and acetate during glucose fermentation while reducing nitrate and sulfur to ammonium and sulfide.	Grabowski *et al*. (2005); Hahnke *et al*. (2016)
*Proteobacteria*	*Xanthobacteraceae*	*Rhodopseudomonas*	Purple non-sulfur bacteria (PNSB) promote plant growth through biocontrol, biostimulation and biofertilisation processes. These processes include enhancing nutrient uptake, generating phytohormones, stimulating immunological responses and interacting with the local microbial ecology. Additionally, PNSB have been observed to help plants cope with abiotic stress by producing endogenous 5-aminolevulinic acid (5-ALA). Furthermore, these bacteria can induce systemic resistance (ISR) in plants, helping them defend against pathogens under biotic stress.	Lee *et al*. (2021)
*Proteobacteria*	*Comamonadaceae*	*Rubrivivax*	*Rubrivivax gelatinosus* TN414 could produce plant growth-promoting substances (1.30 mg/L of NH_4_^+^, 0.94 mg/L of 5-aminolevulinic acid, and 0.65 mg/L of indole-3-acetic acid).	Sakpirom *et al*. (2017)
*Campylobacterota*	*Helicobacteraceae*	*Wolinella succinogenes*	These bacteria could produce succinic acid. Succinic acid has been reported as the major plant growth-promoting compound by stimulation of root hair formation and growth of rice, wheat and barley seedlings.	Tanner *et al*. (1981); Yoshikawa *et al*. (1993)
*Proteobacteria*	*Xanthobacteraceae*	*Blastochloris sulfoviridis*	*B. sulfoviridis*, a purple photosynthetic bacterium, was able to enhance its nitrogen-fixing ability under light-anaerobic conditions.	Huo *et al*. (2024)
*Firmicutes*	*Clostridiaceae*	*Clostridium sensu stricto 1 tertium*	*Clostridium tertium*, classified within *Clostridium sensu stricto 1*, has been identified as a butyrate-producing bacterium with potential probiotic properties. *Clostridium* spp. enhances plant fitness by colonising roots and inducing systemic resistance (ISR). Additionally, bacterial metabolites, including butyric acid, act as ISR determinants, highlighting their potential as bioprotectants.	Jung *et al*. (2023); Wood *et al*. (2025)

**TABLE 5:** Effect of PSB solution treatments on the growth parameters and yield of lettuce, compared to chemical fertiliser and control treatments.

Treatment	Shoot fresh weight (g)	Root fresh weight (g)	Shoot dry weight (g)	Root dry weight (g)	Leaf area (cm)
T1 Control	63.12 ± 7.82C	22.72 ± 4.80C	4.08 ± 0.48C	2.25 ± 0.52C	523.53 ± 28.60
T2 Chemical fertiliser	244.78 ± 14.25A	32.10 ± 5.09B	7.55 ± 0.33A	4.17 ± 1.46AB	504.97 ± 71.17
T3 PSB	253.91 ± 21.10A	28.84 ± 7.68BC	7.54 ± 0.48A	2.91 ± 0.93BC	582.93 ± 28.86
T4 PSB diluted with water at a ratio 1:1	179.93 ± 13.36B	43.70 ± 9.74A	5.96 ± 0.67B	5.13 ± 2.67A	614.07 ± 40.56

*P-*value	< 0.0001^**^	< 0.0001^**^	< 0.0001^**^	0.0128	0.0613

*Notes:* SD = standard deviation. Data in the table are expressed as mean ± SD. Means with same letters in each column are not significantly different at *P* < 0.05, according to Duncan’s new multiple range test.

**TABLE 6:** Chlorophyll A, chlorophyll B and total chlorophyll contents of lettuce leaves (after 56 days of cultivation) under different treatments: T1 (control), T2 (chemical fertiliser), T3 (undiluted PSB solution) and T4 (PSB solution diluted with distilled water at a 1:1 ratio).

Treatment	Chlorophyll A (mg/100 g)	Chlorophyll B (mg/100 g)	Total chlorophyll (mg/100 g)
T1 Control	153.07 ± 0.72BC	72.64 ± 25.83C	225.64 ± 25.19C
T2 Chemical fertiliser	171.77 ± 2.79AB	120.30 ± 24.17B	291.98 ± 23.07B
T3 PSB	195.06 ± 19.52A	164.08 ± 20.19A	359.03 ± 38.97A
T4 PSB diluted with water at a ratio 1:1	137.72 ± 17.60C	145.33 ± 6.27AB	282.97 ± 11.80B

P-value	0.0038	0.0032	0.0021

*Notes:* SD = standard deviation. Data in the table are expressed as mean ± SD. Means with same letters in each column are not significantly different at *P* < 0.05, according to Duncan’s new multiple range test.
